# Pereira’s Materia Medica and Therapeutics, Third Edition

**Published:** 1852-09

**Authors:** 


*Pereira's Materia Medica and Therapeutics, Third Edition.*

This edition, the first vol. of which we have received from Messrs Blanchard & Lea, the enterprising Philadelphia publishers, has been revised and corrected by the author himself. It contains all recent discoveries relating to the subjects treated of in the work, and is in several respects superior to the previous editions. It needs no commendation at our hands. For sale by Keen & Bros., Chicago. J.

